# Household exposure to violence and human rights violations in western Bangladesh (II): history of torture and other traumatic experience of violence and functional assessment of victims

**DOI:** 10.1186/1472-698X-9-31

**Published:** 2009-11-27

**Authors:** Shr-Jie Wang, Mohammad Akramul Haque, Saber-ud-Daula Masum, Shuvodwip Biswas, Jens Modvig

**Affiliations:** 1 Rehabilitation and Research Centre for Torture Victims (RCT), Copenhagen, Denmark; 2 Bangladesh Rehabilitation Centre for Trauma Victims (BRCT), Dhaka, Bangladesh; 3 Dhaka University, Faculty of Social Sciences, Dhaka, Bangladesh

## Abstract

**Background:**

Organised crime and political violence (OPV) and human rights violations have marred Bangladesh history since 1971. Little is known about the consequences for the oppressed population. This study describes the patterns of OPV and human rights violations in a disturbed area of Bangladesh and assesses the physical, emotional and social functioning of victims.

**Methods:**

A total of 236 of selected participants in a household survey in Meherpur district were recruited for a detailed study. Interviews and physical examinations were used to obtain information about history of torture and other cruel, inhuman or degrading treatment or punishment (TCIDTP), and about injuries, pain frequency and intensity. Handgrip strength and standing balance performance were measured. The "WHO-5 Well-being" scale was used to assess the subjective emotional well-being of study participants.

**Results:**

The majority of the reported cases of TCIDTP occurred in 2000-2008, 51% of incidents occurred during winter; 32.0% between 20:00 and midnight. Police involvement was reported in 75% of cases. Incidents took place at victims' homes (46.7%), or at the police station, military camp, in custody or in prison (21.9%). Participants experienced 1-10 TCIDTP methods and reported 0-6 injury locations on their bodies; 77.5% reported having at least two injuries. Less than half of the participants were able to stand on one leg for 30 seconds. Only 7.5% of males aged 25-44 had handgrip strength in both hands exceeding average values for healthy people at the same age. Over 85% of participants scored low (<13) on the 25-point "WHO-5 Well-being" scale. The number of years since the TCIDTP event, pain frequency, the need to quit a job to take care of an injured family member, political involvement, personal conflicts and the fear of neighbourhood violence strongly affected emotional well-being. Good emotional well-being correlated with increased political and social participation.

**Conclusion:**

A detailed picture of characteristics of the victimisation is presented. The participants showed poor emotional well-being and reduced physical capacity. The results indicated that the simple and rapid method of assessment used here is a promising tool that could be used to monitor the quality and outcome of rehabilitation.

## Background

A clinical and functional assessment of the victims is essential when evaluating the effects of organised crime and political violence (OPV). Many studies have suggested that OPV has long-term psychological effects on an individual that persist throughout his or her lifespan. However, research documenting whether OPV results in the deterioration of physical and functional fitness, as well as social relationships, is limited [1-10].

Bangladesh has been a victim of a power struggle between its two major parties since it gained independence. The dominant parties have used armed groups and militias to fight for power [11,12]. The Cingranelli-Richards Human Rights Dataset shows that the people of Bangladesh have been terrorised especially since the 1990s, with increasing numbers of political imprisonments, torture, disappearances and extrajudicial executions [13]. The Political Terror Scale developed by Gibney *et al. *[14] measured the level of human rights violations in Bangladesh as 4 on a scale of 5 since late 1990s, which indicated that the majority of population was affected.

The aims of the present study were to describe the history and pattern of OPV and human rights violations in a border region of Bangladesh, and to measure their consequences for the physical and emotional fitness and social functioning of the victims. It was a pilot study using a simple and rapid method of assessment, which could be further developed for use in countries with limited resources. Such a tool would be valuable for generating a baseline for monitoring the quality and outcome of rehabilitation services.

Our hypothesis in developing the method was that various personal factors, inter-personal relationships, and the extent of political involvement and social participation play a crucial role and interact with the current physical and emotional fitness and functional capacity in victims of OPV and human rights violations. Another hypothesis of the study was that reduced handgrip strength, and poor performance in standing balance measurements - both of which can be simply and inexpensively measured - would be related to having suffered from OPV and human rights violations.

## Methods

Meherpur district, located within the Khulna division and bordered by India's West Bengal to the north, has an area of 716 square kilometres. It is the smallest district in Bangladesh, with an estimated population of 680,000 in 2008. Khulna division has been heavily affected by different forms of violence since the government launched two security operations: Operation Clean Heart in 2002 and Operation Spider Web in 2004.

### Study design and implementation

A protocol was generated based on the World Health Organization (WHO) document "Guideline for conducting community surveys on injury and violence" [15], with a specific focus on OPV-related injury and mortality. The population-based study consisted of two parts: a household survey followed by OPV screening of selected participants in mobile clinics. In the first part of the population-based study, we assessed household experiences of collective exposure to OPV and human rights violations as well as the burden on the families. Data were collected at the individual and family levels. Of the 1,101 households surveyed in Meherpur district from 23 February to 10 March, 2008, over 80% reported exposure to more than two categories of OPV or human rights violations [16].

The second part of the study, reported in this paper, was a retrospective study in which a detailed OPV screening was carried out. The families that participated in the household survey and reported one or more of the following experiences were selected for OPV screening: 1) torture and other cruel, inhuman or degrading treatment or punishment (TCIDTP) (n = 343); 2) sexual harassment, molestation, rape or inserting blunt object into a genital organ and/or rectum (n = 9); 3) arrest and detention without warrant or order (n = 317); or 4) extrajudicial execution of family members (n = 78), perpetrated by members of law enforcement agency. This study used the definition of TCIDTP adopted by the United Nations Convention against Torture and Other Cruel, Inhuman or Degrading Treatment or Punishment. Altogether, 495 families were identified.

The selected families were given vouchers that outlined the aims and the process, including the offer of free medical examination and treatment at mobile clinics. We deployed four mobile clinics: one in Meherpur upazila, and one in Mujibnagar upazila on 12 March, 2008, and two in Gangni upazila on 13 March. The mobile clinics teams consisted of two coordinators, five medical doctors, four physiotherapists, two pharmacists from Dhaka and 12 social workers recruited from a Meherpur-based non-governmental organisation (NGO) Manab Unnayan Kendra, and from Dhaka University.

The 356 people who volunteered to participate were taken in buses to one of the mobile clinics. At the entrance to the clinic the participants were screened again by one of the coordinators. Only people who were victims or secondary victims were included in the study. After the screening process, we excluded 120 participants who were either mentally ill or reported no experience of TCIDTP or others forms of OPV and human rights violations mentioned above. The excluded participants were not interviewed, but they were given a routine medical examination and treatment. Altogether, 236 victims were identified and recruited for the study. Their personal information was collected, including age, sex, education, religion, occupation and area of residence. The questionnaire was developed in English and translated into Bengali. After structured interviews, a physiotherapist or a specially trained interviewer measured height and weight, handgrip strength, and standing balance. A medical doctor performed a physical examination including a routine medical check-up, pulse measurement, and injury examination and provided a consultation. Injuries were recorded on a body map adapted from the Istanbul Protocol: international guidelines for the investigation and documentation of torture. Information about pain frequency and intensity was also obtained.

For the collection of data on body function, activity, participation and environmental factors a 14-point scale was constructed, based on the WHO International Classification of Functioning, Disability and Health [17] (body function: b130, b280, and b760; activity: a340, a450 and a740; participation: p930, p940, p950 and p798; environmental factors: e299, e320, e330 and e470). Subjective difficulties regarding mobility and body functioning were assessed on the following scale: "no", "yes" and "yes with some difficulty".

Physical fitness was measured by assessing muscle strength and equilibrium. Factors that can affect the outcome of these measurements, like weight, height and Body Mass Index (BMI), were also recorded. Under malnutrition conditions, muscular strength decreases before changes in anthropometric and laboratory parameters are noticeable [18-20]. Handgrip strength was measured by a Jamar^® ^hydraulic hand dynamometer according to the recommended procedure of the American Society of Hand Therapists[21]. We used the second handle setting for all participants, and scores were recorded in kilograms. During the measurement, participants were asked to adjust to the right position with their elbows flexed at 90 degrees, grip the dynamometer tightly and hold the grip for three seconds. Within-subject variations were considered and three measurements were taken for each hand. The average and the standard deviation (SD) of highest recorded grip strengths of each participant were calculated. The participants unable to undergo strength measurements because of upper limb deformities were excluded from these measurements.

The equilibrium function of the participants was measured by the standing balance test. The test measures the ability to stand on one leg, making use of all the sensory inputs that contribute to balance, *i.e.*, the central vestibular system, vision, and proprioception (giving information to the brain about leg muscle position). Participants were excluded from testing if they were blind or had amputations or deformities of lower limbs. Participants were asked to stand barefoot on a flat floor on one foot with their eyes open. The physiotherapist/interviewer instructed the participants to adjust the standing position. A silent stopwatch was used to measure the time that each participant could balance on one foot without assistance; participants were asked to stop after 90 seconds.

The "WHO-5 Well-being" questionnaire was used to assess the subjective emotional well-being of study participants. The raw score is calculated by summating the figures of the five answers. Raw scores ranged from 0 to 25. A score of 0 represents the worst possible, and 25 the best possible quality of life. A raw score under 13 is considered indicative of poor emotional well-being.

### Statistical analysis

Data were entered and validated in Microsoft Access 2000 and Epi Info™ 6.04 (CDC Atlanta, USA, 2001). For quality assurance, the data set was checked three times for typing discrepancies and transposition errors. All analyses were carried out using Stata software, version 9.2 (StataCorp LP, Texas, USA, 2003). The 5% significance level (P < 0.05) was used for all analyses. Explanatory variables for personal factors were age group, sex, residence, education, occupation, religion, political party affiliation and level of political and social participation. A generalised linear model was used to study the association between binary outcomes and explanatory variables, adjusted for the effects of other variables.

### Ethical evaluation

The study is compliant with the Declaration of Helsinki and with Danish law. The Bangladesh NGO Bureau gave approval for the study. All of the participants gave informed consent. Treatment was free and severely traumatised cases were referred to the Bangladesh Rehabilitation Centre for Trauma Victims. The confidentiality and safety of participants is our primary concern. All the procedures used were designed to protect the privacy of the participants and the confidentiality of the information. It can be a risk for the oppressed population to participate in such a study; therefore, both the Bangladesh Rehabilitation Centre for Trauma Victims and the Task Force against Torture will be following up on the legal and health needs of victims once our study is completed. The sponsor had no role in the study design, data collection, analysis, and interpretation, and writing of the report.

## Results

### Socio-demographic profile

The sample population characteristics are presented in Tables 1 and 2. The mean age of 236 participants was 42.2 with a SD of 14 years (range 12-81) with no difference between men (78%) and women (22%). Only four participants were under 18 years old and 20 were over 65 years old. Nearly 50% of participants had no formal education at all.

**Table 1:** Social demographic profile of study participants, n = 236

Social demographic data	Variables	No. of victims (%)
Upazila (sub-district) of Meherpur district	Gangni upazila	64 (27.2)
	Mujibnagar upazila	89 (37.9)
	Meherpur Sadar upazila	82 (34.7)
	Missing	1 (0.4)

Education level	None	114 (48.3)
	Primary	63 (26.7)
	Secondary	37 (15.7)
	College or university	10 (4.2)
	Post-graduate	4 (1.7)
	Koran school	1 (0.4)
	Others	6 (2.5)
	Missing	1 (0.4)

Religion	Muslim	229 (97.0)
	Hindu	6 (2.5)
	Missing	1 (0.4)

Participation in a religious or spiritual activity within 7 days preceding the survey	No	89 (37.9)
	Yes	146 (62.1)
	Missing	1 (0.4)

Occupation	Not working	6 (2.5)
	Household work	48 (20.3)
	Agriculture, fishing, animal husbandry or hunting	110 (46.6)
	Business	30 (12.7)
	Service, journalist or teacher	3 (1.3)
	Other	38 (16.1)
	Missing	1 (0.4)

Involved in political party	None	138 (58.5)
	Awami League	59 (25.0)
	Bangladesh Nationalist Party (BNP)	29 (12.3)
	Jamaat-e-Islami Party	10 (4.2)

Level of political affiliation	Supporter	174 (73.7)
	Member	34 (14.4)
	Activist	5 (2.1)
	Leader	2 (0.8)
	Missing	21 (8.9)

Often hold a meeting at home or attend a meeting in the community	No	212 (89.8)
	Yes	24 (10.2)

Have personal, financial or political conflict with other people	No	68 (28.8)
	Yes	168 (71.2)

Have ever participated in a demonstration, a strike or a human rights rally	No	131 (55.5)
	Yes	105 (44.5)

Have relative or friend working with law enforcement agency	No	136 (57.6)
	Yes	100 (42.4)

Have relative or friend involved in illegal activity	No	196 (83.0)
	Yes	40 (17.0)

Have good friends in whom you confide and who help you	No	136 (57.6)
	Yes	100 (42.4)

**Table 2:** Health indicators of study participants

Health indicators	Male (n)	Female (n)	Total
**Sex**	183	52	235

**Age group**	**Male (n)**	**Female (n)**	**Total (%)**

5-14	0	1	1 (0.4)
15-24	13	2	15 (6.5)
25-34	30	12	42 (18.1)
35-44	63	18	81(34.9)
45-54	39	10	49 (21.1)
55-64	19	5	24 (10.3)
≥65	17	3	20 (8.6)

**WHO-5 Well-being questionnaire**	**Male (n)**	**Female (n)**	**Total (%)**

Score ≥13	27	5	32 (14.2)
Score <13 (poor emotional well-being)	149	44	194 (85.4)

**Body size**	**Male**	**Female**	

Height (cm): mean (min-max)	163.1 (145-180)	152.5 (140-169)	
Weight (kg): mean (min-max)	55.0 (35-82)	51.6 (26-78)	

**Body Mass Index (BMI: kg/m^2^)**	**Male (n)**	**Female (n)**	**Total (%)**

BMI<16.5	14	2	16 (7.0)
16.5≤ BMI<18.5	25	9	34 (15.0)
18.5≤ BMI<23	97	20	117 (51.5)
23≤ BMI<27	35	14	49 (21.6)
BMI≥27	5	6	11 (4.8)

**Handgrip strength mean (kg)**	**Male**	**Female**	

Mean (min-max) for the right hand (kg)	30.6 (0-55)	23.4 (12-41)	
Mean (min-max) for the left hand (kg)	29.5 (0-50)	20.3 (0-45)	

**Standing balance mean (seconds)**	**Male**	**Female**	**Total**

Mean (95% CI) for the right leg	46.5 (41.4-51.6)	38.2 (30.1-46.4)	44.5 (40.1-48.8)
Mean (95% CI) for the left leg	46.0 (40.9-51.1)	39.8 (31.6-48.1)	44.4 (40.1-48.8)

**Standing balance performance**	**Male (n)**	**Female (n)**	**Total**

Right leg >30 seconds	94	24	118
Right leg ≤ 30 seconds	82	28	110
Left leg >30 seconds	95	25	120
Left leg ≤ 30 seconds	81	27	135

**Pain intensity within 2 weeks**	**Male (n)**	**Female (n)**	**Total (%)**

No pain	3	0	3 (1.3)
Light pain	17	8	25 (11.0)
Moderate pain	133	37	170 (74.9)
Severe pain	22	7	29 (12.8)

**Pain frequency within 2 weeks**	**Male (n)**	**Female (n)**	**Total (%)**

Constant pain (all the time)	42	14	56 (25.2)
Periodic pain (one or more times a week)	85	26	111(50.0)
Occasional pain (less than once a week)	44	11	55 (24.8)

### Political activity and social life

The level of political involvement was considered because during the household survey, the affiliation of a family member to a political party was found to be a risk factor for victimisation [16]. Overall, 174 participants (80.9%) were "party supporters" but 117 of them did not name any specific political party. There were 59 (12.3%) who named the Awami League and 29 (12.3%) the Bangladesh Nationalist Party (BNP). Only 34 participants (15.8%) claimed to be members of a political party, five (2.3%) were activists (two for the Awami League and three for the BNP) and two (0.9%) were local political leaders (one for the Awami League and the other for the BNP). There were 24 participants (10%) who said they often held meetings at their homes or attended meetings in the community, and among them, 15 were party members and three were activists. Among the party supporters interviewed, 69.5% supporters of the Awami League, 55% of supporters of the BNP, and 60% of supporters of the Jamaat-e-Islami Party have participated in some type of demonstration, a strike or a human rights rally.

Over two-thirds of participants reported having personal, financial or political conflicts with other people. Over half of participants had been out to visit their friends within 7 days. When they were asked about their fear of violence in the community, 22.1% of participants said they had no fear of violence in the community, but 7.5% said they were often afraid, and 5.0% that they were always afraid of violence.

### History of torture and other traumatic experience of violence

**Figure 1:**
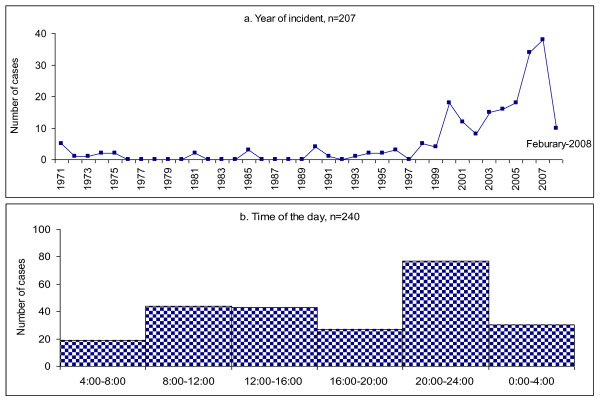
**Trend and patterns of incident reporting**.

The earliest cases of TCIDTP reported by the participants occurred as early as 1971 when the liberation war against Pakistan took place (Figure 1a). Only a few cases were reported during the first fifteen years of post-war Bangladesh from 1975 to 1990. The number of reported TCIDTP cases remained small and stable in 1991-1995 but doubled in 1996-1999. Since 2000, the reported numbers have increased sharply. On average, 6.3 years (range 0-37) passed between the time of TCIDTP and the present survey. Two of the participants who had been abused in 1971 were abused again in subsequent years.

**Figure 2:**
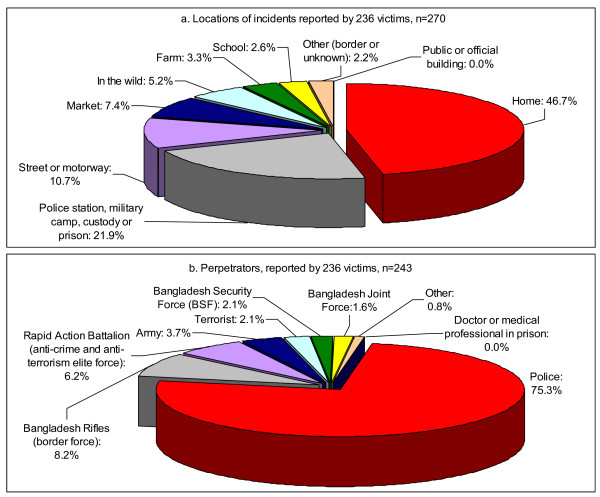
**Locations and types of perpetrators**.

The results demonstrated that 118 participants (51.1%) were subjected to TCIDTP during the 4.5-month cool and dry winter season (mid October to February), while 76 participants (32.9%) recalled the incident being during the 4-month hot and humid summer season (March to June) and 37 participants (16.0%) during the 3.5-month rainy monsoon season (July to mid October). Nearly one third of participants alleged that they were subjected to TCIDTP between 20:00 and midnight, which is the peak hour (Figure 1b). Events often occurred at the residence of the victims (46.7%), and among them, 40% occurred between 20:00 and midnight. Perpetration also occurred in closed environments, *i.e.*, in a police station, in a military camp, in custody or in prison (21.9%). Only 10.7% incidents occurred in open areas on streets or motorways (Figure 2a). The participants endured a total of 719 TCIDTP events.

The mean number of TCIDTP methods reported by each participant was 3.0 with a SD of 2.0. The maximum was 10 (reported by two members of Awami League). Around 20% of the participants (n = 46) were subjected to at least five methods. Among them, 19 were from the village called Shibpur and eight were from the village of Monakali; both of which are located along the Bangladesh-Indian border in Mujibnagar upazila. The five most frequently applied methods of TCIDTP were: 1) kicking; 2) beating with a blunt object; 3) falanga (beating the soles of the feet); and 4) threats to the family and mock execution; 5) other psychological torture (Table 3). In addition, sixteen participants (12 males and four females) experienced sexual harassment or forced sexual contact. One such participant was the local political leader of Awami League, who was also subjected to 10 methods of TCIDTP.

**Table 3:** Torture methods reported by the study participants

Rank	Torture method	No. of victims (%)
1	Being kicked	171 (23.8)
2	Other psychological torture	143 (19.9)
3	Being beaten or hit with a blunt object	94 (13.1)
4	Falanga (beating on the soles of the feet)	65 (9.0)
5	Threats to family, mock execution	46 (6.4)
6	Strangulation	43 (6.0)
7	Immersion in water or suffocation	34 (4.7)
8	Being blindfolded	30 (4.2)
9	Forced position	24 (3.3)
10	Needle pricking	23 (3.2)
11	Electric shock	16 (2.2)
12	Sexual harassment, molestation, rape or inserting blunt object into a genital organ and/or rectum	16 (2.2)
13	Other	13 (1.8)
14	Burn	6 (0.8)
	Total	724

The police were the major perpetrators, being responsible for 183 cases (75.3%), while 20 acts of TCIDTP (8.2%) were committed by the Bangladesh Rifles (Bangladesh border force), 15 (6.2%) by the Rapid Action Battalion (anti-crime and anti-terrorism elite force) and five (2.1%) by terrorists (Figure 2b). Among five victims of terrorists, two were also abused by the police and three were secondary victims. Among the 16 cases of sexual harassment or forced sexual contact, 12 involved the police, one involved the Rapid Action Battalion, one involved terrorists, one involved the Bangladesh Rifles, and one involved the Bangladesh Joint Force. The results showed that Meherpur Sadar police used torture methods more frequently than the Gangni and Mujibnagar police, i.e. they were responsible for 43 out of 65 falanga episodes, 11 out of 16 electric shock episodes and 10 of the 16 episodes of sexual harassment and forced sexual contact. It was also demonstrated in the household survey that more families living in Meherpur Sadar reported being subjected to TCIDTP [16]. Only 59 (25.1%) participants had sought legal support in relation to their cases

### Health condition of victims

The number of reported injury locations ranged from 0 to 6: 33.5% of participants reported having one injury location, and 66.5% reported having at least two (Table 4). The three most frequently injured areas were the lower back and abdomen (128 reported injuries), the legs (100 reported injuries), and the chest (58 reported injuries). The participants were at increasing risk of having leg or knee injury if they had been subjected to falanga (odds ratio [OR] = 2.10, 95% CI = 1.28-3.67, P < 0.01) or to being kicked (OR = 2.7, 95% CI = 1.32-5.53, P < 0.01). There is an association between falanga and sole injury (OR = 4.26, 95% CI = 1.45-12.49, P < 0.01). Pain experience is shown in Table 2. During the two weeks preceding the survey, 25.2% of participants had experienced constant pain and 50.0% periodic pain.

**Table 4:** Injury reporting by the study participants

Numbers of reported injuries on the body map	No. of victims (%)
1	76 (33.5)
2	100 (44.1)
3	32 (14.1)
4	15 (6.6)
5	2 (0.9)
6	2 (0.9)
Missing	9
Total	236

**Body mapping of injury**	**No. of injury (%)**

Forehead	9 (2.0)
Head	19 (4.2)
Shoulder	40 (8.8)
Chest	58 (12.8)
Lower back and abdomen	128 (28.2)
Arms	23 (5.1)
Legs	100 (22.0)
Ankles	26 (5.7)
Toes	2 (0.4)
Fingers of both hands	10 (2.2)
Palms of both hands	4 (0.9)
Total	419

Height and weight were measured for 228 participants (Table 2) (eight participants missed the measurement). BMI is defined as an individual's body weight divided by the square of his or her height. The mean BMI was 20.7 with a SD of 3.0 (range 13-29) for males and was 22.2 with a SD of 3.9 (range 12.9-31.2) for females. We used the cut-off values for being overweight (23.0≤ BMI<27.0) and obese (BMI≥27.0 kg/m^2^) recommended for a Southeast Asian population by Singaporean and Indian researchers [22,23]. These values are somewhat lower than the global values. Around 22% of males and 21.6% of females were considered underweight (16.5≤ BMI<18.5) and malnourished (BMI<16.5). Only 8.5% of males were overweight (23.0≤ BMI<27.0), and no man was obese (BMI≥27), while 19.6% and of females were overweight and 2.0% obese. There are significant age and gender effects on BMI. Female participants generally have a higher BMI than males of the same age group (coef = 0.43, 95% CI = 0.07-0.79, P < 0.05).

All participants were tested for their handgrip strength. For males, the mean strength was 30.6 with a SD of 10.7 kg for the right hand and 29.9 with a SD of 10.6 kg for the left hand. For females, the mean strength was 23.4 with a SD of 6.0 kg for the right hand and 20.3 with a SD of 7.1 kg for the left hand. For 147 out of 231 participants (63.6%), right hand strength dominated left hand strength. We found that five participants had no strength in either hand, and two had no strength in their left hands. Lacking the national average for the general population in Bangladesh, we compared the mean handgrip strength of male participants aged 25-44 years old with that of a reference group of 100 healthy males aged 27-42 years in West Bengal: that of 50 office workers was 43 kg and that of 50 metal workers performing intensive manual labour was 41 kg [24]. The mean handgrip strength of 93 male participants in our study, aged 25-44 years, was 31.8 with a SD of 10.5 kg for the right hand and 30.0 with a SD of 10.9 kg for the left hand. Among them, eight (8.6%) and 17 (18.3%) had a handgrip strength equal to or above the mean handgrip strength of healthy male office workers and the male brass metal workers. Only 7 of them had both handgrip strengths equal to or above 41 kg.

In total, 229 participants were able to take the standing balance test. The mean duration of standing balance on the right foot was 44.5 seconds (95% CI: 40.1-48.8) and the mean duration for the left foot was 44.4 seconds (95% CI: 40.1-48.8). The difference between the mean duration of standing balance between male and female participants is shown in Table 2. Only 102 participants (44.5%) were able to stand on either foot for 30 seconds while 34 (14.9%) could maintain their balance standing on one foot or the other for 30 seconds. A number of 93 participants (40.6%) were unable to stand in a balanced position on either foot for 30 seconds.

Standing balance is negatively correlated with age over 55 years (OR = 0.37, 95% CI: 0.21-0.65, P < 0.001), but there is no association with gender. Obese people (BMI≥27) have more difficulty to maintain a standing balance (OR = 0.16, 95% CI = 0.03-0.81, P < 0.05), as compared to people of normal size (18.5≤ BMI<23.0) or underweight and malnourished people (BMI<18.5). After adjusting for age and BMI and the cluster effect of villages (prevalence of annual injury rate and violence-related injury was related to the level of exposure to OPV varied by villages) [16]), we found a strong negative association between standing balance and the number of injuries (OR = 0.10, 95% CI = 0.01-0.90, P < 0.05) and leg and knee injury (OR = 0.57, 95% CI = 0.35-0.96, P < 0.05).

One of the questions we asked participants was whether they had difficulty in walking to the other side of their village, or whether they needed help. The distances involved depended on which village a participant lived in. Odds ratios for good standing balance adjusted for age, BMI and the cluster effect of village were 2.94 (95% CI = 1.33-6.52, P < 0.01) for people who reported no difficulty in walking to another side of their village, and 2.67 (95% CI-1.30-5.47, P < 0.01) for people who needed assistance to walk this distance. The self-reported difficulty of walking thus correlates with the objective measurement on standing balance. No statistical association was found between standing balance and the number of years that had passed since the incidence of TCIDTP or between standing balance and the self-reported pain intensity and frequency during the two weeks preceding the survey, or to being subjected to falanga and being kicked.

Of the 225 participants who completed the "WHO-5 Well-being" questionnaire (one participant was missing), 194 (85.84%) scored less than 13 (Table 2). A generalised linear model was used to evaluate the results and study the relationship between subjective emotional well-being, political involvement or social participation and personal factors (Table 5). Poor emotional well-being was not associated with being more than 55 years old, sex or education level. We did find a correlation between emotional well-being and pain frequency: people who had occasionally or periodically experienced pain within 2 weeks preceding the survey were at lower risk of a poor emotional well-being than people who suffered constant pain (OR = 0.03, 95% CI = 0.00-0.19, P < 0.001 and OR = 0.09, 95% CI = 0.01-0.82, P < 0.05, respectively). Also, participants were less likely to have poor emotional well-being if more years had passed since being subjected to TCIDTP (OR = 0.82, 95% CI = 0.72-0.93, P < 0.005) or if they were more involved in social or political activity: the odds ratio was 0.05 (95% CI: 0.01-0.34, P < 0.005) for people who had participated in a demonstration, a strike or a human rights rally. A strong statistical difference is shown between different levels of political involvement: the activists are less likely to have poor emotional well-being (OR = 0.03, 95% CI = 0.01-0.19, P < 0.001) than the general party supporters. Involvement in a conflict with other people (OR = 21.33, 95% CI: 5.81-78.30, P < 0.001), frequent fear of violence in the neighbourhood (OR = 3.23, 95% CI: 1.64-6.36, P < 0.001) and the experience of stopping work to take care of an injured family member (OR = 14.21, 95% CI = 1.31-154.00, P < 0.05) were all strongly associated with increasing vulnerability to poor levels of emotional well-being.

**Table 5:** Standing balance performance, emotional well-being and its association with personal factors and health condition

Variables (Both legs stand more than 30 seconds)	No	Yes	OR (95% CI)	P value
BMI<16.5	8	8	1	-
16.5≤ BMI<18.5	21	13	0.51 (0.19-1.36)	0.180
18.5≤ BMI<23	57	59	0.71 (0.29-1.75)	0.461
23≤ BMI<27	32	17	0.45 (0.16-1.25)	0.125
BMI≥27	9	2	0.17 (0.03-0.81)	< 0.05
Male	95	81	1	-
Female	31	21	0.96 (0.35-2.60)	0.930
Age<55	95	88	1	-
Age≥55	30	12	0.37 (0.21-0.65)	< 0.001

Injury mapping (adjusted for age and BMI)	No	Yes	OR (95% CI)	P value

0 injury	3	6	1	-
1-2 injuries	89	80	0.21 (0.02-2.20)	0.19
≥3 injuries	35	16	0.10 (0.01-0.90)	< 0.05
Without leg and knee injury	66	66	1	-
With leg and knee injury	61	36	0.57 (0.35-0.96)	< 0.05
Can not walk to another side of village	34	11	1	-
Able to walk to another side of village without assistance	51	51	2.94 (1.33-6.52)	< 0.005
Need assistance to walk to another side of village	38	35	2.67 (1.30-5.47)	< 0.005

Pain (adjusted for age and BMI)	No	Yes	OR (95% CI)	P value

No pain	1	2	1	-
Light pain	14	11	0.31 (0.01-15.50)	0.56
Moderate pain	105	65	0.22 (0.00-10.19)	0.44
Severe pain	23	7	0.12 (0.00-6.24)	0.29
Constant pain (all the time)	43	14	1	-
Periodic pain (one or more times a week)	63	48	1.84 (0.76-4.46)	0.18
Occasional pain (less frequent than once a week)	35	20	1.24 (0.49-3.15)	0.65

Years passed since TCIDTP incident (adjusted for age and BMI)	No	Yes	OR (95% CI)	P value

0	6	4	1	-
1-2 years	39	32	0.92	0.902
3-5 years	25	22	1.28	0.732
6-10 years	27	19	0.70	0.672
>10 years	14	13	2.16	0.415

**Variables (WHO-5 Well-being <13, poor emotional well-being)**			**OR (95% CI)**	**P value**

Political party member vs. general party supporter			0.53 (0.77-3.77)	0.522
Activist vs. general party supporter			0.03 (0.01-0.19)	< 0.001
Often attend meeting or hold meeting at home			1.27 (0.12-13.04)	0.839
Have participated in demonstration, a strike or a human rights rally			0.05 (0.01-0.34)	< 0.005
Have conflict with other people			21.33 (5.81-78.30)	< 0.001
Number of years passed since TCIDTP incident			0.82 (0.72-0.93)	< 0.005
Periodic pain vs. constant pain			0.09 (0.01-0.82)	< 0.05
Occasional pain vs. constant pain			0.03 (0.00-0.19)	< 0.001
Frequency of fear of violence in the community			3.23 (1.64-6.36)	< 0.001
Stop working to take care of an injured family member for a period			14.21 (13.1-154.00)	< 0.05
Education level			0.88 (0.62-1.23)	0.444
Age≥55			3.40 (0.56-20.62	0.183

We categorised the victims into four different groups based on the methods of TCIDTP they were subjected to. The "basic level" consists of 1) beating with a stick or a blunt object, 2) blindfolding and 3) kicking. The "basic + psychological torture level" indicates the "basic level" plus psychological torture. Victims who were subjected to torture at a "basic level" and in addition to any severe torture method were grouped as "basic + severe torture cases". The victims who were subjected to torture at a "basic level" and in addition to psychological torture plus any of the severe torture methods were grouped as "all". Out of 224 victims (two participants missed reporting any TCIDTP method), 46 (20.5%) had been subjected to the "basic level" of TCIDTP while 41 (18.3%) and underwent "basic + psychological torture" and 35 (18.3%) underwent "basic + severe torture". A further 102 of the 224 victims (45.4%) were subjected to "all" TCIDTP methods. No statistical association was found between the types/levels of TCIDTP methods victims were subjected to and either their physical performance (hand grip strength and standing balance), or their emotional well-being.

## Discussion

The present study provides a detailed clinical and functional assessment of a sample of 236 participants in a border area in Bangladesh who reported during a household survey [16] that they had been subjected to OPV and human rights violations during the past 38 years. We used a combination of physical examination and simple physical tests, a questionnaire and interviews to record the history and present situation of the participants and to assess their social and physical functioning and well-being.

The participants' reports did not only provide information about individuals, but about the wider history of OPV and human rights violations. The earliest reported incident in our study was in 1971. The first arrest reported in the patient records of the Bangladesh Rehabilitation Centre for Trauma Victims was also in 1971. There may have been incidents before the liberation war, but there is some doubt as to whether the victims are still alive. In our study, only a few incidents were reported from 1971 to 1990. From 1991 to 1995 there were still relatively few, but the frequency doubled in 1996-1999, and since 2000, the reported numbers have increased sharply. The years 1971, 1990-91, 1996-97 and 2000-01 are crucial in the history of OPV and human rights violations in the whole of Bangladesh. In these years, there was a high level of political tension and violence; this pattern is shown in our study and also reported by other institutions [11,12,25,26]. There were general elections in Bangladesh in 1991, 1996 and 2001, which were wrecked by pre and post-election violence. Over 80% of the participants in our study claimed to be "party supporters" but only a few of them revealed which political party they supported. Therefore we were unable to do further analysis to test our hypothesis that the supporters of an opposition party were more likely to be victimised as the political tension increased sharply in the year of general elections.

Human rights violations including the use of TCIDTP have intensified in the last five years in Meherpur district since Operation Spider Web was launched, and violence and crime have been aggravated. Since 2006, the enforcement of strict emergency regulations by a care-taker government has increased the brutality of violent confrontations.

The results also showed a seasonal pattern. The number of reported cases was three times higher during the winter season than during the rainy season. It is clear that the police and the military are hampered by the flooding and uncomfortable conditions during the rainy season.

From the results of our study it appeared that the police were the major perpetrators. Individuals could be attacked by armed militias associated with party politics, but such incidents were not reported here. Such individuals were not likely to present in the mobile clinic, because the recruitment criteria were primary or secondary victims subjected to any of four categories of OPV and human rights violations perpetrated by the members of law enforcement agency. Mitchell (2004) pointed out that the degree of political control that the political authorities exercise over a law enforcement agency varies across time and political system. It is plausible that if individuals in a law enforcement agency have goals independent of those of the authority, or have private interests, this may influences the choice, level and method of violence [27].

The survey provided detailed information about the methods used by the perpetrators. A previous study in Sri Lanka suggested that perpetrators generally use readily available materials as TCIDTP instruments [28]. Methods gain popularity if they cost nothing, are available anywhere at any time, can achieve maximum effect, and rarely leave clear evidence, e.g., falanga (beating the soles of the feet) or being kicked.

Forced sexual contact and sexual abuse are also frequently-used forms of torture. It was reported that all of the female refugees and one-third of the male refugees from Bangladesh examined at the Centre for Torture and Trauma Survivors in Stockholm alleged that they had been raped [29]. In our study, few participants reported that they had been sexually harassed, abused or raped. However, owing to the social stigma involved, victims may not admit to being raped or sexually abused by members of a law enforcement agency unless they are far away from the perpetrators and from their own community. This helps to explain the low number of cases identified in our study. Among them, there were more males (n = 12) than females (n = 4). Rape and sexual abuse of males is not rare: it was shown that 22% of inmates in Nebraska, USA in 1996 [30] and 21% Tamil detainees in Sri Lanka reported at least one episode of forced sexual contact [31]. Up to now, the epidemic character of using male rape and sexual abuse as a weapon in the conflict setting [32-34] and in closed environments, *i.e. *detention and prison, has been neglected by the authorities. A low level of control of corruption in the administration is likely to provide the members of law enforcement agency with a wealth of opportunities for hidden actions including the perpetration of sexual violence [35]. It is also plausible that in the society in which homosexuality is not approved of, the setting of detention and prison allows the individuals of law enforcement agency who seek for particular sexual interest to conceal their actions.

Very little is known about the physical and emotional consequences and social functioning of an oppressed population experiencing collective exposure to OPV and human rights violations. The rehabilitation of TCIDTP victims has been mainly based on clinical experiences seen from an illness perspective. For people who continue to live in their communities, it is most important that they should be able to maintain daily life after being traumatised. A basic level of muscle strength and physical mobility is required, simply to be able to cope with the activities of daily life.

In our study we assessed muscular function by measuring handgrip strength, which is required in many daily activities in a rural area. Hand dynamometer testing is recommended to determine the loss of handgrip strength [36-38]. This method is widely used for outcome documentation after injuries of the upper extremities [39], as a functional index of nutritional status, and for determination of impairment [40]. Our study was the first to use the method to investigate loss of muscle strength in a survey of a population exposed to massive OPV and human rights violations. Because standard normative values for healthy adults for all age groups in Bangladesh or in South Asia are not available, we used values for young and middle aged male adults in West Bengal [24]. Our survey participants showed lower muscle strength in their dominant hands. However, we did not determine whether the reduced muscle strength was the consequence of the traumatic experience, and a further study with a control group is needed.

Our study was also the first to assess standing balance performance in an oppressed population. Impaired lower extremity performance is associated with reduced physical activity levels, which may contribute to subsequent disability in elderly persons [41,42]. Standing balance is related to physical fitness and consequently to social life [43,44]. Many participants reported lower back, leg and knee injuries, which can affect standing balance. We found evidence that there was an association between objective measurements of standing balance performance and self-reported walking performance, which also indicated the reliability of subjective difficulty measured and reported by the victims. The standing balance test is a simple rapid assessment tool and could be considered as an instrument to be used routinely in the initial functional assessment procedure of victims of torture or other forms of violence. We recommend conducting a comprehensive balance and mobility assessment when standing balance performance of a victim is poor even after adjustment for age and BMI.

The measures used to test muscle strength and physical mobility in our survey are rapid and inexpensive, so they are appropriate for use in countries with limited resources. Such tests can be used to produce essential information for purposes of diagnosis and prognosis, as well as for prevention and rehabilitation. The results could also contribute to quantifying the economic burden in terms of disability and manpower lost due to OPV and human rights violations, and their association with poverty in a country such as Bangladesh.

A person's functioning does not only depend on muscular strength but also on emotional factors. We used the brief questionnaire "WHO-5 Well-being" to estimate the percentage of victims with poor subjective emotional well-being, and to examine the association of subjective emotional well-being with social participation. Emotional, physical, and social vulnerability as a consequence of being abused is related to the development of post-trauma stress disorder and other mental disorders. The inter-personal and inter-family conflicts are high in the study area. In order to develop programmes which can help to reduce the damage to emotional health and to prevent its harsher effect on mental health, we need to understand what factors may empower victims to cope with their vulnerability.

One factor that we considered was active participation in political or social movements. The effects of political and social participation on well-being are complex. On the one hand, in the household survey we found that if a family member was affiliated to a political party or participated in a demonstration, a strike or a human rights rally, this was a risk factor for victimisation [16]. On the other hand, the present study found that participation in a political or social movement was linked to improved emotional well-being; it could strengthen people's self-confidence and restore their interest in social justice and the environment. Many trauma victims have a poor self-image and low self-esteem. Organising or taking part in a demonstration, a strike or a human rights rally allows participants to share and release their feelings of stress and frustration, to express their anger in a collective voice, and to bond with others and create alliances. Participation can empower the victims and thus improve their emotional well-being. These interactions demonstrate the complexity of the factors that determine emotional well-being; on the one hand, the patterns of political or social participation affect the emotional well-being of trauma victims, but, on the other hand, their emotional well-being also determines the personal, social, and behavioural competence in relationships and the capacity to deal constructively with a challenging or difficult situation [45,46].

### Limitations and strengths

Our findings concerning perpetrators and years of perpetrations were consistent with reports from other international institutions [11,12,25,26] and also from a local human rights organisation, Odhikar. A medical examination that found traces of injury is also a validation of the oral reports. We used simple physical tests to confirm the reliability of subjective difficulty in walking reported by the study participants.

One limitation was that owing to logistical and political constraints, we did not recruit people without prior TCIDTP experience as a healthy control group while taking into account neighbourhood effect on OPV or human rights violations. In addition, there was inevitably some risk of bias in the recruitment of the study group. The health-seeking behaviour of the individuals concerned is one possible source of bias. We do not know why some who had vouchers decided not to come to the mobile clinic. Some of them may have been so severely depressed that they lacked any motivation to come - and others may have seen no need to interrupt their work to come to the clinic. It is also possible that there was some bias because a few participants exaggerated their injuries and pain experiences in order to receive more treatment. Memory bias does exist, but the main increase in OPV and human rights violations in this area took place within the last 10 years, and ten-year recall is considered reliable [47].

We had hoped to use the results as a baseline for a large-scale intervention in this community, and to repeat the measurements afterwards to monitor the quality and outcome of rehabilitation. We had already developed plans for various interventions including setting up a platform, Victim Association, where the survivors can talk about their fears and stigmas and re-construct their self-images, and which will also serve as a place for social participation and political empowerment. The members should assist the Victim Association to raise the community awareness and spread knowledge about human rights by organising various community activities. Unfortunately, these cannot immediately be realised for unexpected reasons

## Conclusion

This study presents trends and patterns of OPV and human rights violations in the Meherpur district. Generally, the oppressed population showed poor emotional well-being and a reduced level of physical fitness. Among study participants, good subjective emotional well-being correlated with increased political and social participation. To monitor the quality and outcome of rehabilitation, the simple and rapid methods used here could be developed into a valuable tool for initial assessment. Further studies are also needed to establish reference values in the general population and oppressed populations within this complex setting.

## List of abbreviations

BNP: Bangladesh Nationalist Party; BRCT: Bangladesh Rehabilitation Centre for Trauma Victims; BMI: Body Mass Index; OPV: organised crime and political violence; OR: odds ratio; NGO: non-governmental organisation; RCT: Rehabilitation and Research Centre for Torture Victims; SD: standard deviation; TCIDTP: torture and other cruel, inhuman or degrading treatment or punishment; WHO: World Health Organization.

## Competing interests

The authors declare that they have no competing interests.

## Authors' contributions

SJW participated in the design of the study, conducted the field work, analysed and interpreted data and drafted the manuscript. MAH, SDM and SB participated in the coordination in the field, managed and supervised the data collection. JM participated in the conception of the work, helped to draft the manuscript and revised it critically. All the authors have read and approved the final version of the manuscript.

## Pre-publication history

The pre-publication history for this paper can be accessed here:

http://www.biomedcentral.com/1472-698X/9/31/prepub
